# Four-Port 38 GHz MIMO Antenna with High Gain and Isolation for 5G Wireless Networks

**DOI:** 10.3390/s23073557

**Published:** 2023-03-28

**Authors:** Ahmed A. Ibrahim, Wael A. E. Ali, Moath Alathbah, Ayman R. Sabek

**Affiliations:** 1 Electronics and Communications Engineering Department, Minia University, Minia 61519, Egypt; 2 Department of Electronics & Communications Engineering, College of Engineering and Technology, Arab Academy for Science, Technology and Maritime Transport (AASTMT), Alexandria 21937, Egypt; 3 College of Engineering, King Saud University, Riyadh 11451, Saudi Arabia; 4 School of Engineering, Cardiff University, Cardiff CF24 3AA, UK

**Keywords:** MIMO antenna, MIMO performance, high gain, 5G wireless communications, FSS structure

## Abstract

In this paper, a 38 GHz 4-port multiple-input multiple-output (MIMO) antenna with considerable isolation and gain enhancement for 5G applications is introduced. The suggested antenna element is a monopole antenna composed of a circular patch with a rectangular slot etched from it and a partial ground plane is used to extend the desired frequency to operate from 36.6 GHz to 39.5 GHz with a center frequency of 38 GHz. The high isolation is achieved by arranging the four elements orthogonally and adding four stubs to reduce mutual coupling between elements at the desired frequency bands. The gain improvement is also introduced by placing a frequency selective structure (FSS) which is designed at the same frequency bands of the antenna under the suggested MIMO antenna to act as a reflector. The proposed four-element MIMO with the FSS prototype is built and tested in order to confirm the simulated results. The suggested antenna operated from 37.2 GHz to 39.2 GHz with an isolation of less than 25 dB across the obtained frequency range. The peak gain of the antenna is enhanced from 5.5 dBi to around 10 dBi by utilizing the FSS structure; furthermore, the back radiation is enhanced. The MIMO performance is validated by extracting its parameters and comparing with the simulated results. The results extracted from the simulation and the measurement show satisfactory matching along with the target band, indicating that the proposed structure could be used for 5G communications.

## 1. Introduction

The fifth-generation (5G) communications are distinguished by three distinct characteristics: universal connectivity, extremely low latency, and extraordinarily high data transmission rates [1,2]. This new fifth-generation (5G) communication network, with high-capacity and high-rate data transmissions, allows 5G to be integrated with the internet of things (IoT) technology [3,4]. Because of their large bandwidth, the millimeter-wave (mm-Wave) bands corresponding to frequencies ranging from 30 GHz to 300 GHz have received a lot of attention. When compared to existing wireless technologies, mm-Wave communications have various advantages: extremely wide bandwidths, larger spectrum resources, and small element sizes [5,6]. The Federal Communications Commission (FCC) makes mm-wave spectrum operation above 24 GHz available for 5G wireless in four bands: 24.75–25.25 GHz, 37.6–38.6 GHz, 47.2–48.2 GHz, and 50.4–51.4 GHz [7].

Antenna design is one of the most complicated issues for future 5G cellular connectivity. Various experts have been working on 5G antennas that resonate at a frequency of 38 GHz [8,9,10,11,12,13]. In [11], a four-element MIMO antenna resonates at 38 GHz with a peak gain of 7.6 dBi, and an isolation greater than 20 dB is introduced. In [12], a unique single layer for a 5G (MIMO) antenna with isolation greater than 20 dB and a peak gain of 7.7 dBi is presented. A compact design of a 4 × 4 massive MIMO antenna that resonates at 38.9 GHz with a defective ground structure enhances isolation between segments is proposed in [13]. Thus, by presenting a MIMO system with considerable isolation between antenna ports, the total system performance can be enhanced in terms of larger data rate and capacity, and lower multipath effect [14,15,16,17].

Gain enhancement can be accomplished by utilizing several techniques such as employing array configuration [18,19,20], an artificial magnetic conductor (AMC) [21,22,23], and an appropriately constructed FSS reflector to create an in-phase reflection throughout the full bandwidth [24,25,26,27,28,29]. In [18], a compact 1 × 4 broadband dual-polarized (DP) array is presented with end-fire radiation and with enhanced gain from 4 dBi to 7.1 dBi. In [19], A novel dense dielectric (DD) patch array antenna operating at 28 GHz is presented to improve gain by more than 16 dBi. In [20], broadband printed dipole antenna and arrays for (5G) wireless cellular communication networks are presented, with gain enhancement from 4.5 dBi to 12 dBi using an 8-element array.

A feasible configuration of a slotted bowtie antenna with an AMC structure is introduced in [21]. In [22], a high-gain and wideband MIMO antenna that resonates at 28 GHz is introduced with an AMC array to increase the antenna gain to 10 dBi. In [24], the use of a 2D transmission FSS structure to improve the gain to 10.3 dB is discussed. In [25], a novel design of a double dielectric resonator antenna (DRA) with a gain enhancement of 3.16 dBi is achieved by using FSS. In [26], four ports circular polarization (CP) antenna are proposed for a 30 GHz MIMO system with an FSS superstrate to enhance the gain by around 1.5 dBi.

In this paper, a 38 GHz MIMO antenna composed of highly isolated four elements with an FSS structure is designed and simulated using HFSS for 5G applications. To achieve the anticipated 5G frequency ranges, the circular patch is cut by a rectangular slot. Furthermore, to accomplish the high isolation properties of the MIMO configuration, the four elements of the recommended antenna with a size of 25.95 × 25.95 × 0.238 mm^3^ are joined and positioned orthogonally with four stubs. The MIMO testing findings in terms of impedance and radiation characteristics are extracted to investigate the desired performance of the MIMO antenna. Moreover, the MIMO diversity parameters such as envelope correlation coefficient (ECC), diversity gain (DG), channel capacity loss (CCL) are also extracted. The novelty of this work is the design of a simple 4-port antenna operated at 38 GHz applications. Second, the antenna achieved isolation between ports around more than 25 dB, which is suitable for this application. Third, the antenna has an enhanced gain of 10 dBi with the help of the FSS structures. Fourth, the antenna has a suitable overall size and diversity performance which is recommended for 38 GHz applications.

## 2. Design Procedures of Single Monopole Antenna

**Figure 1:**
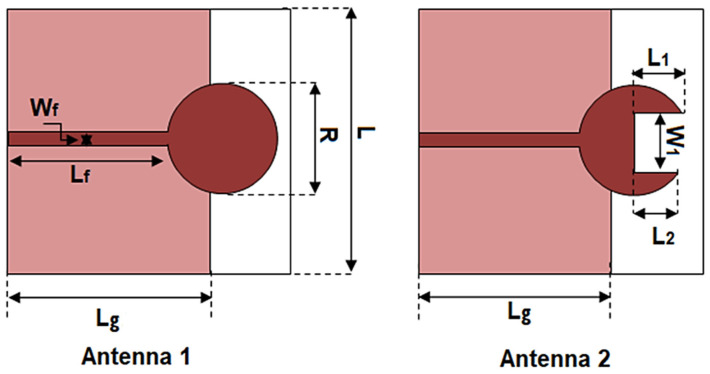
The evolution of a 38 GHz circular patch antenna.

**Figure 2:**
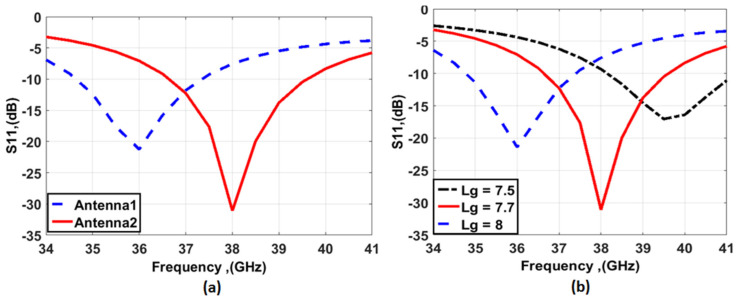
The circular patch antenna results (**a**) S_11_ outcomes of the antennas (**b**) The effect of the ground length (Lg).

The single antenna design phases are shown in Figure 1. It is simulated on a Rogers RT 4003 substrate with a thickness h = 0.203 mm, and dielectric constant ε_r_ = 3.55, with an overall size of L × L = 12 × 12 mm^2^. First, a circular patch monopole antenna is designed with a diameter R = 4.94 mm, a partial ground plane with a length (Lg1) of 8 mm, and a 50 Ω feedline with a width (Wf) of 0.4 mm and a length (Lf) of 7 mm is introduced as a start point of the design. As shown in Figure 2a, the blue dashed curve (antenna 1) resonates at a fundamental mode of 36 GHz with bandwidth extended from 34.8 GHz to 37.3 GHz. To achieve the suggested frequency at 38 GHz, antenna 2 is introduced. By etching a rectangular slot with W1 = 2.2 mm, L1 = 2.45 mm, and L2 = 2.35 mm, Lg = 7.7 mm is introduced to obtain a frequency range extended from 36.6 GHz to 39.6 GHz as depicted in Figure 2a (red solid). Additionally, the effect of the ground length (Lg) on the antenna 2 performance is shown in Figure 2b. When the ground length (Lg) equals 7.5 mm, the antenna is operated at 39.5 GHz. By increasing the Lg to 7.7 mm, the operated frequency is shifted down to 38 GHz. Finally, by increasing it to 8 mm, the antenna is operated at 36 GHz.

**Figure 3:**
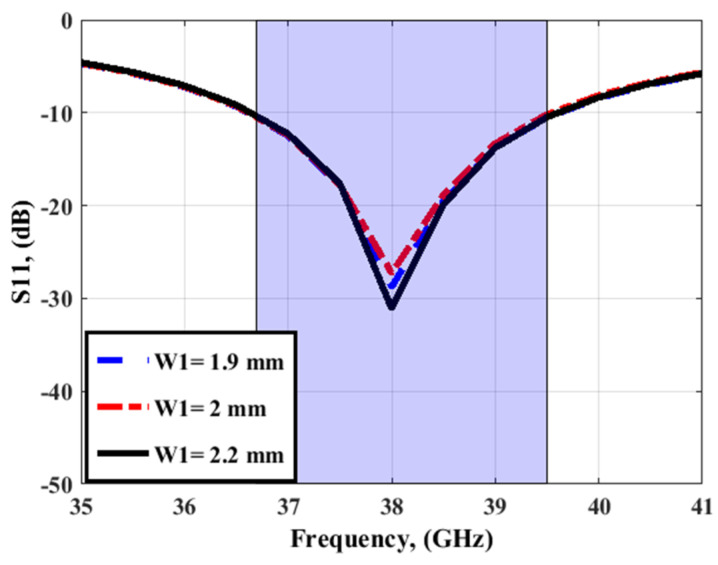
The W1 effect on the antenna performance.

**Figure 4:**
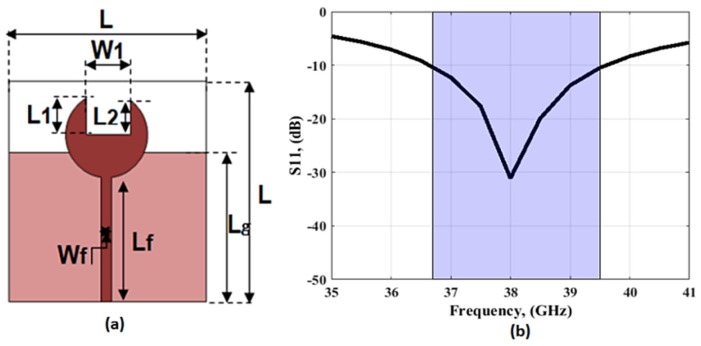
The suggested 38 GHz antenna (**a**) 2D configuration (L = 12 mm, L1 = 2.45 mm, L2 = 2.35 mm, W1 = 2.2 mm, Wf = 0.4 mm, Lf = 7 mm, and Lg = 7.7 mm) (**b**) The S_11_ result.

From the design procedures above, antenna 3 is recommended for producing the desired 38 GHz frequency bands. By using the HFSS simulator, a parametric study is performed to obtain the optimized width (W1) of the rectangular slot as shown in Figure 3. It is seen that W1 can affect the depth of the S_11_ level while the bandwidth of the antenna was not affected. The 2D layout with the optimized dimensions is shown in Figure 4a and the simulated S_11_ outcomes are displayed in Figure 4b. The simulated outcomes are accomplished frequency bands from 36.5 GHz to 39.5 GHz with deep S_11_ levels of −30 around 38 GHz in the recommended band.

## 3. Four-Port MIMO Antenna and Its Parametric Analysis

**Figure 5:**
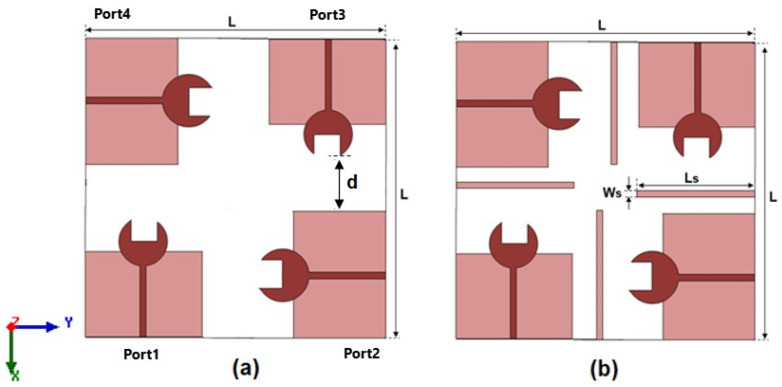
The 2D configuration of the 4-port MIMO antenna (**a**) without stubs and (**b**) with stubs.

This section discusses the structure of a 4-port MIMO antenna and the method used to enhance isolation between elements. As indicated in Figure 5, the MIMO antenna is discussed in two designs with an (L × L) size of 25.95 × 25.95 m^2^. The single antenna unit discussed in the previous section is copied three times and placed orthogonally to each other as depicted in Figure 5a, and the detachment (d = 3 mm) between the four elements is the same as depicted in Figure 5a. To enhance the isolation between ports, four stubs with a width of Ws = 0.5 mm and a length of Ls = 12 mm are utilized as depicted in Figure 5b. 

**Figure 6:**
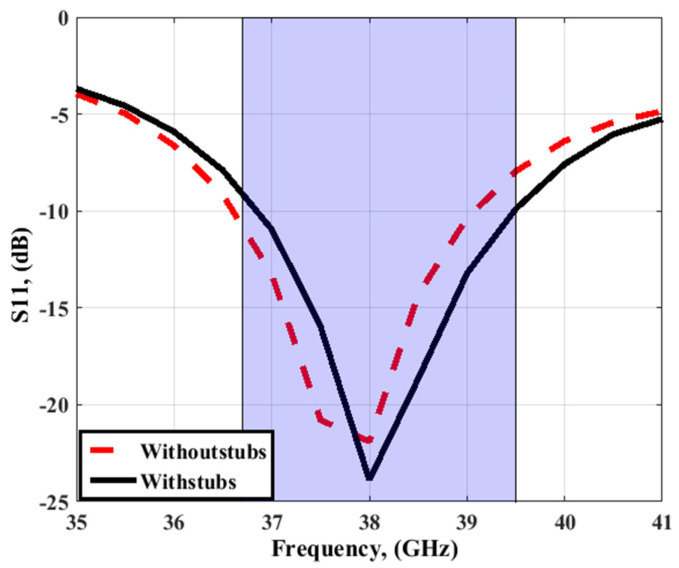
The simulated S_11_ with/without stubs.

**Figure 7:**
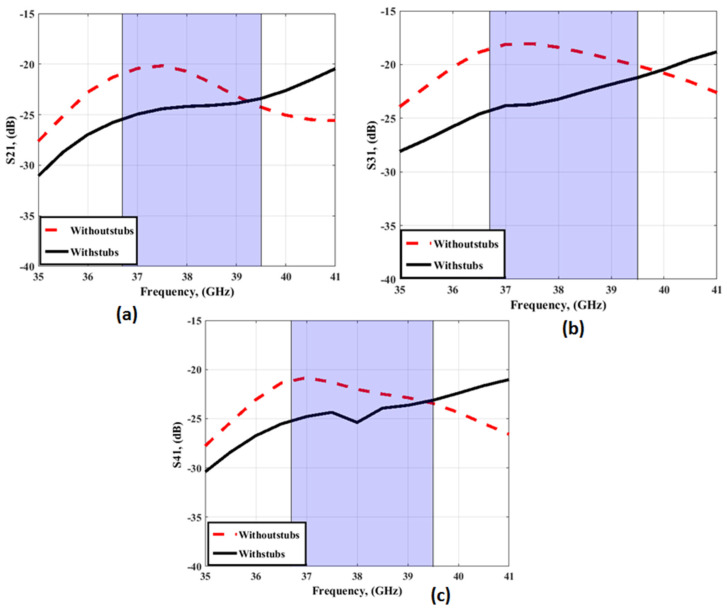
The simulated transmission coefficients with/without stubs (**a**) S_21_ (**b**) S_31_ (**c**) S_41_.

Figure 6 and Figure 7 depict the simulation results to compare the two designs and demonstrate the influence of adding the four stubs on the separation between antenna elements. As depicted in Figure 6, S_11_ with and without stubs shows that the two antennas operated at approximately the same frequency bands. The return loss between antenna elements (S_21_/S_31_/S_41_) is improved by around 5 dB by introducing the four stubs, especially at 38 GHz, which validates the MIMO antenna with stubs to be used instead of without stubs in this paper.

**Figure 8:**
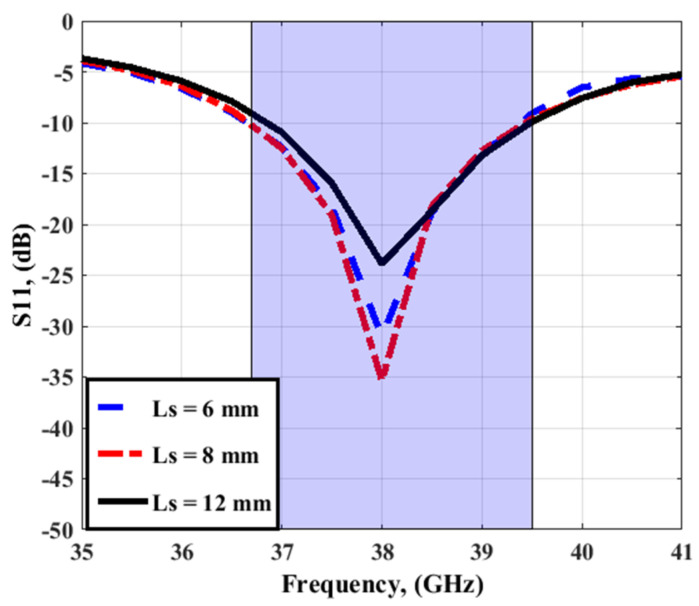
The effect of the Ls on the antenna performance.

**Figure 9:**
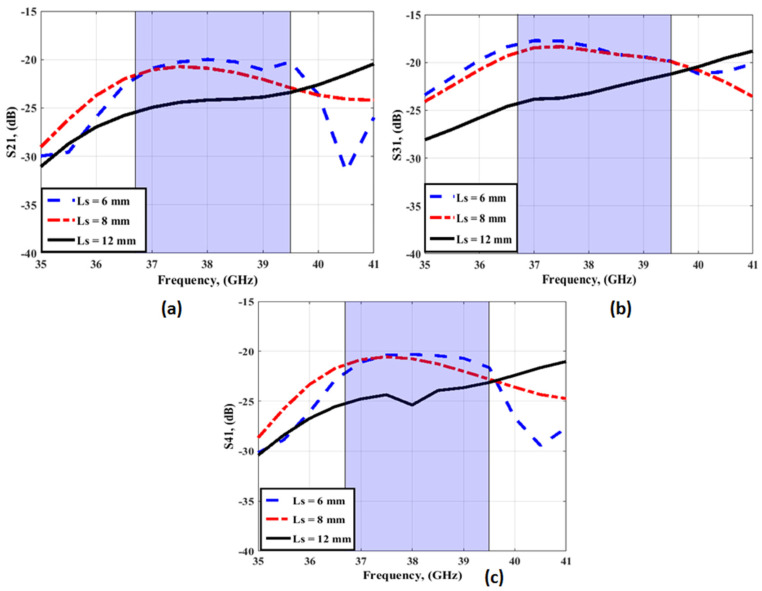
The simulated transmission coefficients with changing Ls (**a**) S_21_ (**b**) S_31_ (**c**) S_41_.

For achieving the high performance of the four-element MIMO antenna, parametric studies were performed. The parametric study to show the influence of changing the length of isolation stubs (Ls) on antenna performance operates as depicted in Figure 8 and Figure 9. As shown in Figure 8, Ls length affects the antenna matching while the bandwidth of the antenna is the same. However, it affected the isolation between ports as shown in Figure 9. The isolation between ports is improved as the length of stubs (Ls) is increased from 6 to 12 mm, as shown in Figure 9. Therefore, the optimum length of isolation stubs is 12 mm, and its width Ws = 0.5 mm.

**Figure 10:**
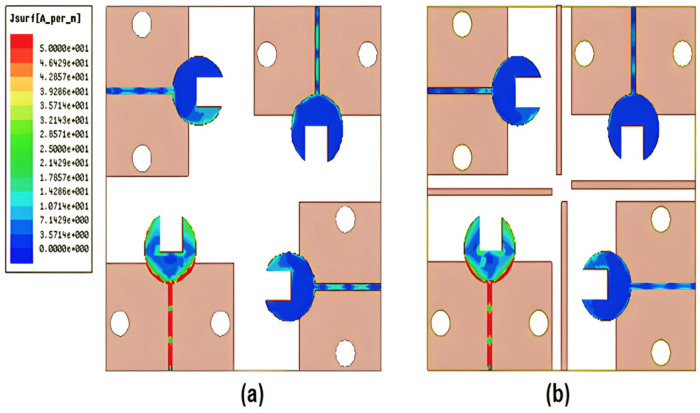
The surface current distribution at 38 GHz (**a**) without stubs (**b**) with stubs.

Figure 10 shows the simulated surface current distribution for the 4-port MIMO antenna with/without stubs at 38 GHz. As shown, the current distribution in the case of the presence of the stubs has a small amount of current going to the other port compare to without a stub, which validates the high isolation between ports as shown in Figure 10b.

**Figure 11:**
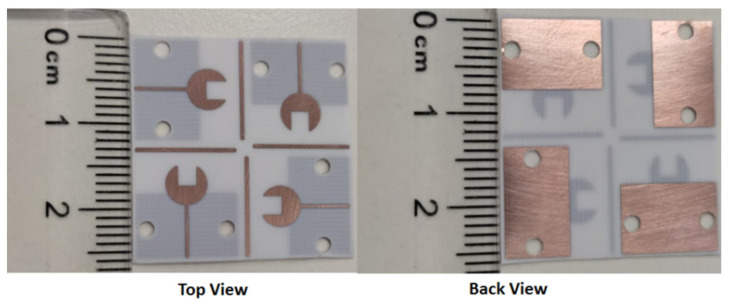
The fabricated prototype photo (Top/Back views) of the 4-port MIMO antenna.

Figure 11 shows the fabricated prototype photo (Top/Back views) of the 4-port MIMO antenna. The 4-port MIMO antenna has a total size of 25.95 × 25.95 × 0.238 mm^3^ and is joined and positioned orthogonally with four stubs.

**Figure 12:**
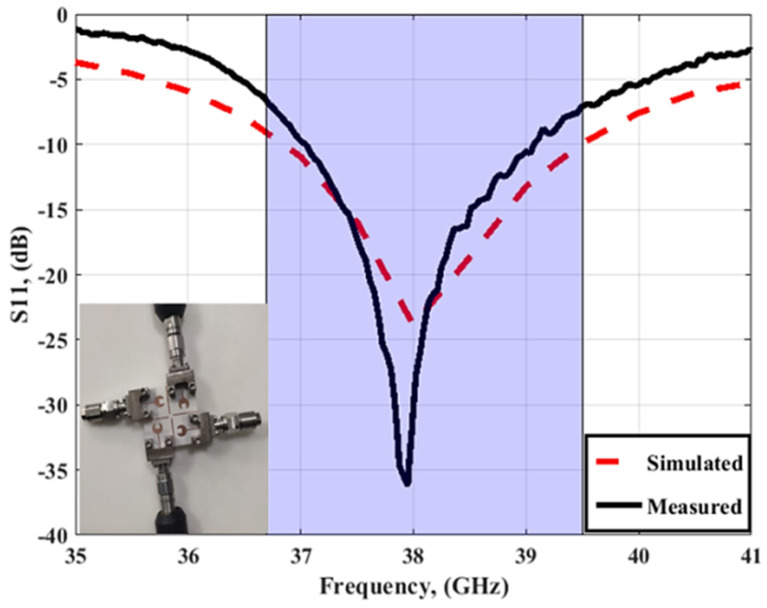
The simulated and measured S_11_ outcomes of the MIMO antenna at port 1.

**Figure 13:**
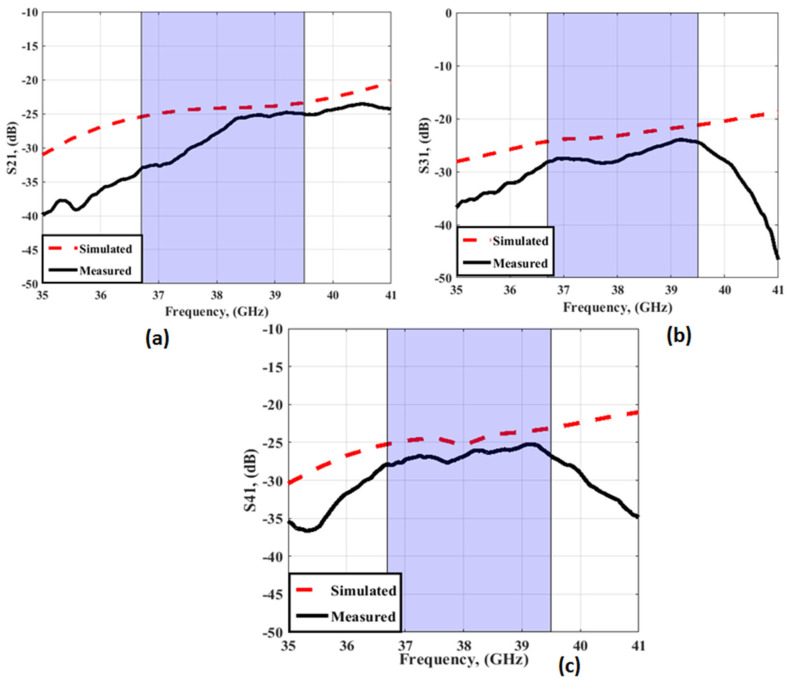
The simulated transmission coefficients of the MIMO antenna at port 1 (**a**) S_21_ (**b**) S_31_ (**c**) S_41_.

Figure 12 and Figure 13 depict the simulated as well as the measured outcomes for the suggested MIMO configuration that demonstrates the accepted reflection coefficient and the transmission coefficients between ports. The simulated outcomes have a frequency band from 36.7 GHz to 39.5 GHz, the S_11_ reaches −24 dB, and the coupling between antenna elements (S_21_/S_31_/S_41_) is <−22 dB. On the other hand, the tested results which are extracted using (R&S ZVA 67 VNA) are from 37 GHz to 39 GHz with S_11_ reaching −36.2 dB, and the isolation between ports (S_21_/S_31_/S_41_) is <25 dB. There is a slight difference between the two outcomes due to fabrication and measurement tolerances that cannot be resolved.

**Figure 14:**
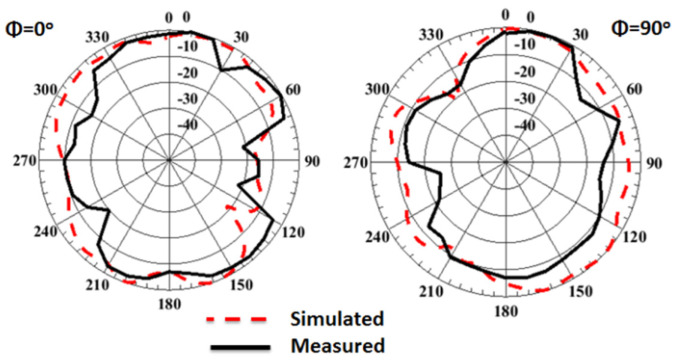
The normalized radiation patterns outcomes of 4-port MIMO antenna at port 1 and 38 GHz.

Figure 14 depicts the normalized radiation pattern outcomes of the 4-port MIMO antenna at port 1 and 38 GHz when the other three ports are connected to 50 Ω. The antenna has a semi bidirectional radiation pattern at φ = 0° and semi omnidirectional pattern at φ = 90°. Finally, due to manufacturing and testing tolerances, there is a minor difference between the two outcomes.

## 4. FSS

This section investigates the characteristic of the FSS unit cell and studies the effect of the cell size on the MIMO antenna performance.

**a.**  **The FSS Unit cell**

**Figure 15:**
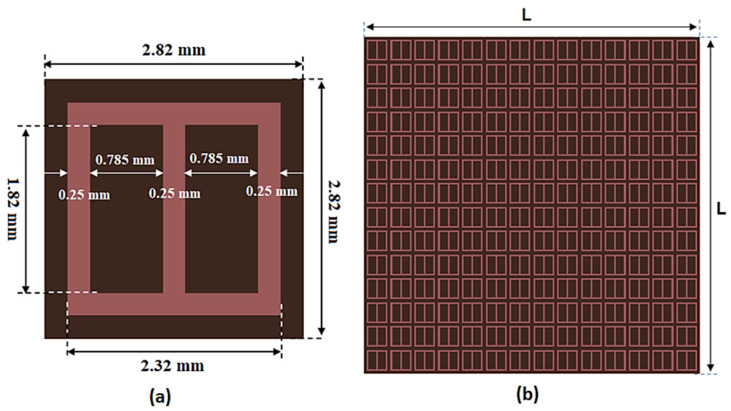
The FSS structures (**a**) Unit cell and (**b**) the suggested FSS array cells.

**Figure 16:**
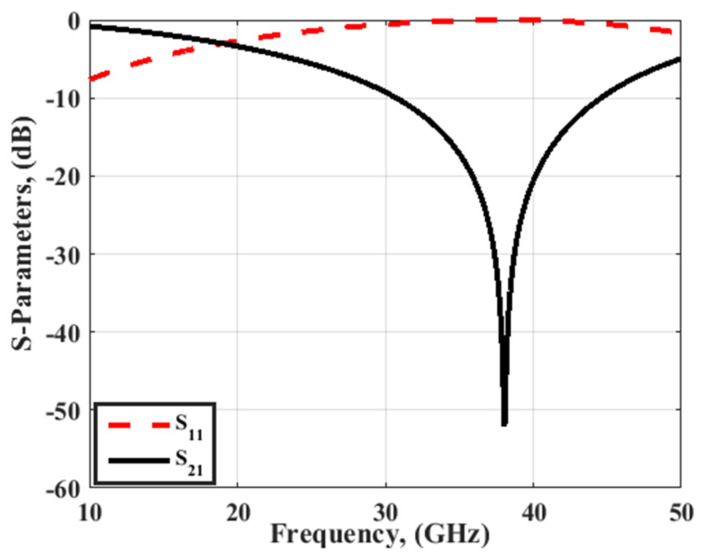
The FSS unit cell S-parameters results.

As discussed in the literature review, The FSS is considered one of the techniques utilized to improve the antenna gain. By placing the FSS array under the antenna, it can be worked as a reflection structure to reflect the back-radiation and enhance the radiation characteristics of the antenna. The FSS unit cell and the suggested FSS array are shown in Figure 15. Rogers’ 5880 substrates with a 0.5 mm thickness, ε_r_ = 2.2, and a total size of 2.82 mm × 2.82 mm are utilized in the simulation and fabrication. A copper layer of 0.035 mm in thickness with two rectangular slots is added on top of the substrate, as shown in Figure 15a, and there is no copper layer on the back of it. The FSS unit cell S-parameters outcomes are illustrated in Figure 16. The outcomes show that the FSS achieves band-stop features from 30 GHz Up to 45 GHz with an S_21_ response lower than −10 dB, and the lowest level is introduced at 38 GHz. Additionally, S_11_ touches 0 dB which means the FSS can be used as a reflector.

**b.**  **Antenna attached with FSS array**

**Figure 17:**
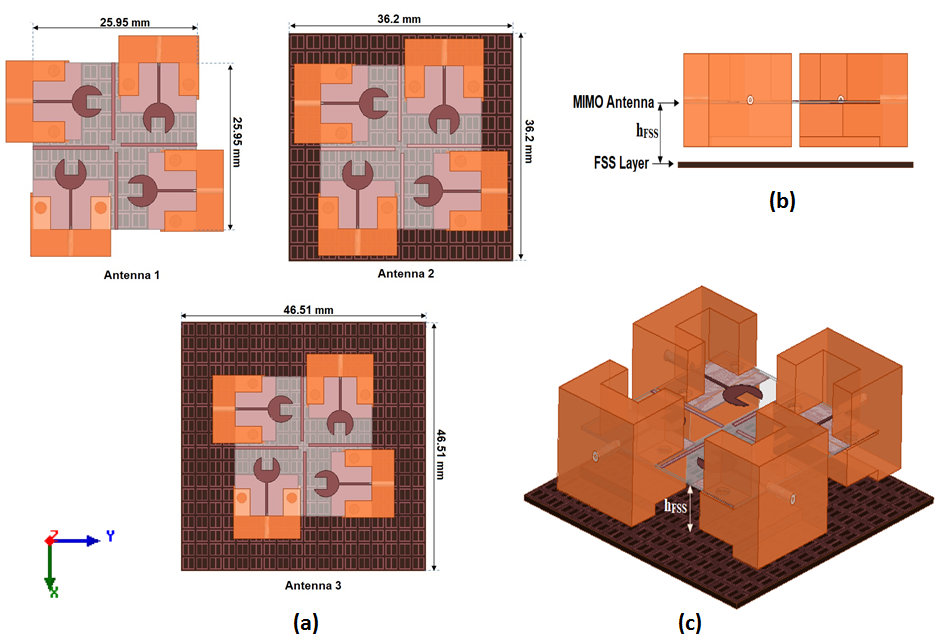
The proposed MIMO antenna attached with FSS structures (**a**) Three antennas with different FSS cell sizes (**b**) side view (**c**) 3D view.

**Figure 18:**
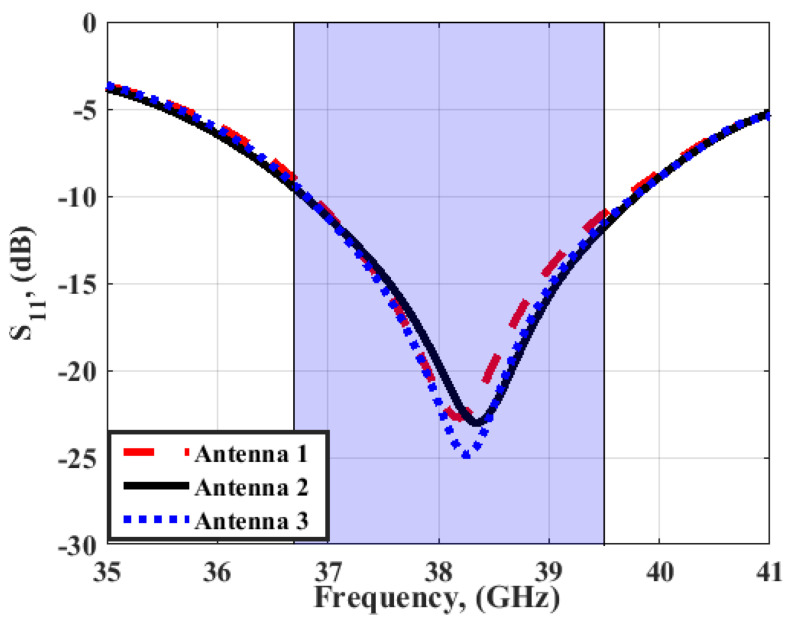
The simulated S_11_ outcomes of the MIMO antenna at port 1 at different FSS cell sizes.

**Figure 19:**
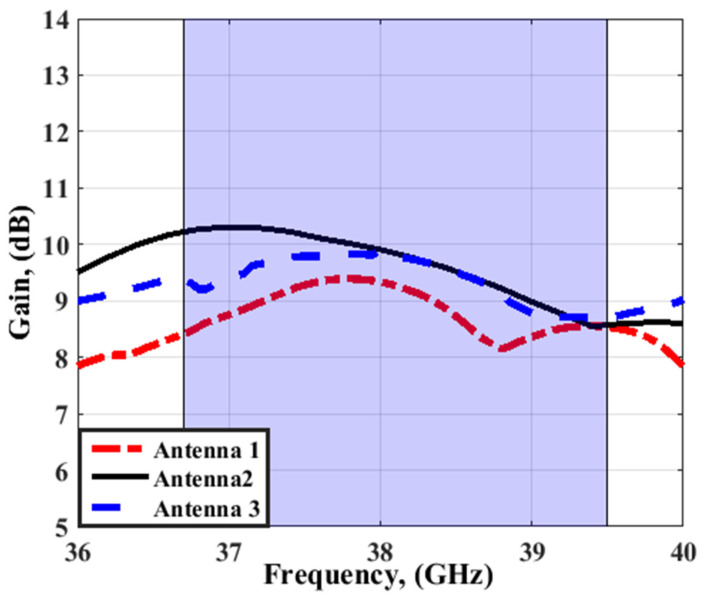
The simulated peak gain of the MIMO antenna at port 1 at different FSS cell sizes.

To investigate the effect of the FSS cells array on the MIMO antenna performance, such as S_11_ and peak gain, the suggested MIMO antenna is attached with different FSS cells size and placed at a distance of 5 mm, as shown in Figure 17. Antenna 1, antenna 2, and antenna 3 have 10 × 10, 14 × 14, and 18 × 18 FSS cells sizes, respectively. It is clear that by increasing the array size, the reflection coefficient and the bandwidth are almost the same as shown in Figure 18. However, the peak gain of the antenna is affected. When the FSS array equals 10 × 10, 14 × 14, or 18 × 18 cells, the gain of the antenna has around 9 dBi, 10 dBi, and around 10 dBi at 38 GHz, respectively, as shown in Figure 19. It means that when increasing the cell size, the gain is almost the same. Thus, for reducing the antenna size, the FSS arrays with 14 × 14 cells are utilized.

## 5. The Proposed Four-Port MIMO Antenna

**Figure 20:**
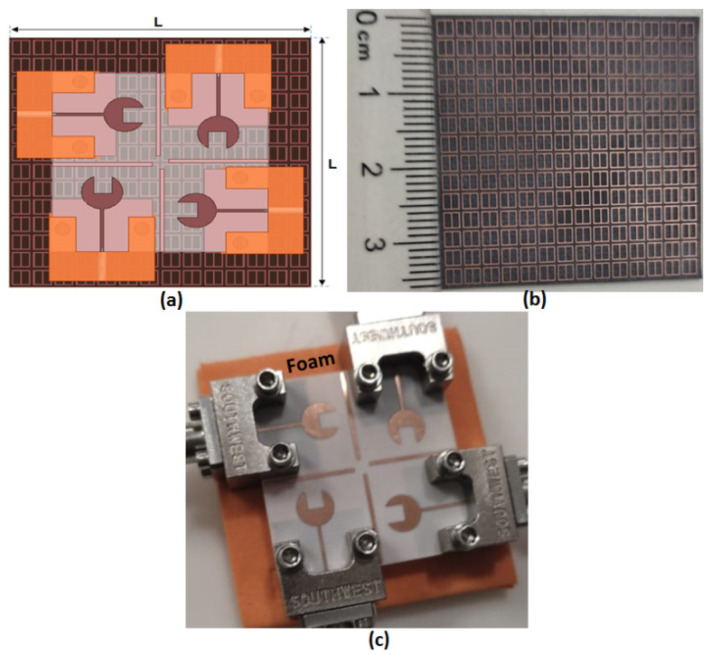
The suggested 4-port MIMO antenna (**a**) 2D layout (**b**) The fabricated photo of 14 × 14 FSS cells (**c**) The fabricated prototype loaded with FSS cells.

**Figure 21:**
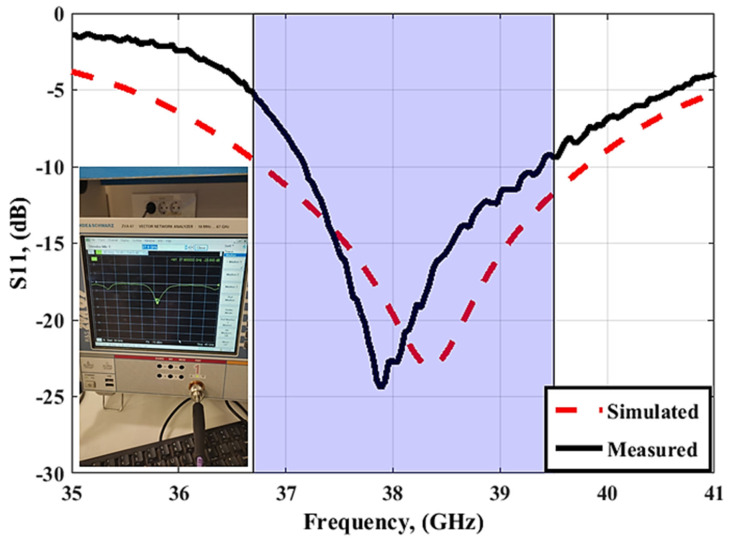
The simulated and measured S_11_ outcomes of the MIMO antenna with FSS at port 1.

**Figure 22:**
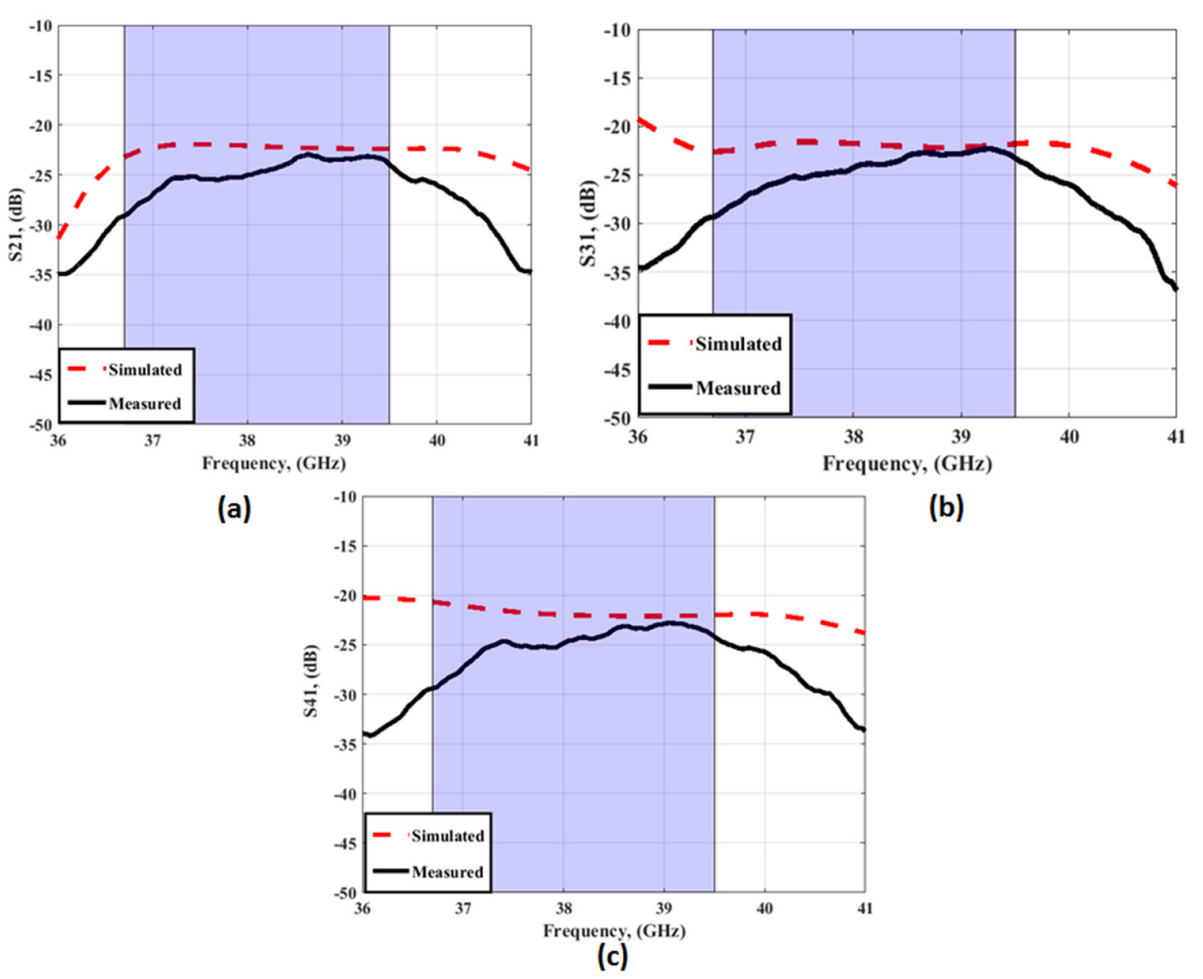
The simulated transmission coefficients of the MIMO antenna with FSS at port 1 (**a**) S_21_ (**b**) S_31_ (**c**) S_41_.

Depending on the previous discussion to improve the suggested antenna gain, a 14 × 14 FSS array (36.2 mm × 36.2 mm) is added under the proposed MIMO. The suggested 4-port MIMO antenna with a 14 × 14 FSS cell is fabricated as shown in Figure 20. A foam layer with ε_r_ = 1.03 of polystyrene and 5 mm thickness is added between the MIMO antenna and the FSS cells, as shown in Figure 20c. The simulated and measured outcomes for S_11_ and isolation between elements (S_21_/S_31_/S_41_) are shown in Figure 21 and Figure 22. 

The simulation result shows that the proposed antenna cover the band from 36.7 GHz to 39.5 GHz, with S_11_ reaching −21 dB, and the isolation between antenna elements (S_21_/S_31_/S_41_) is <20 dB. While the tested outcomes are accomplished by frequency bands from 37.2 GHz to 39.2 GHz, S_11_ reaches the maximum level of −24.4 dB at 38 GHz, and the isolation between antenna elements (S_21_/S_31_/S_41_) equals 25 dB. The simulated and measured outcomes have a good match within the operating band (38 GHz). However, due to the fabrication and measurement tolerances, there is a difference between the two results outcomes.

**Figure 23:**
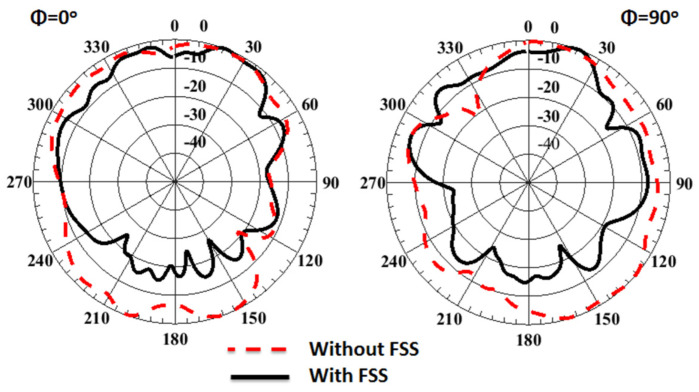
The normalized radiation patterns outcomes of 4-port MIMO antenna at port 1 at 38 GHz with/without FSS.

The normalized simulated radiation patterns outcomes of the 4-port MIMO antenna at port 1 at 38 GHz with/without FSS are illustrated in Figure 23. It is seen that the FSS cells reduced the back loop of the antenna and enhanced the gain of the antenna.

**Figure 24:**
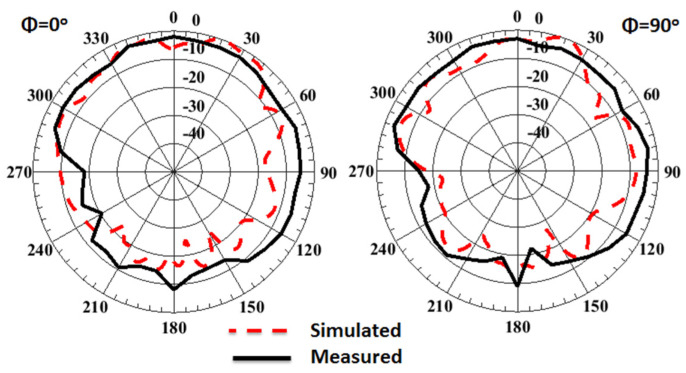
The normalized radiation pattern outcomes of the 4-port MIMO antenna with FSS at port 1 and 38 GHz.

**Figure 25:**
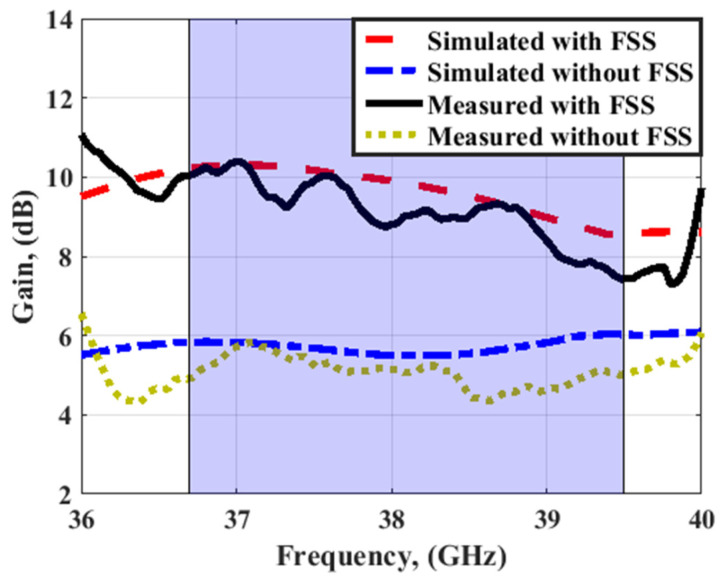
The simulated and measured peak gain outcomes of the MIMO antenna with/without FSS at port 1.

The normalized radiation pattern outcomes of the 4-port MIMO antenna with FSS at port 1 at 38 GHz are shown in Figure 24. By placing the FSS under the MIMO antenna, the maximum power is concentrated in one direction, resulting in gain enhancement, and reducing the back radiation compared to the structure without FSS. The simulated and tested peak gain outcomes of the MIMO antenna with/without FSS at port 1 are illustrated in Figure 25.

**Figure 26:**
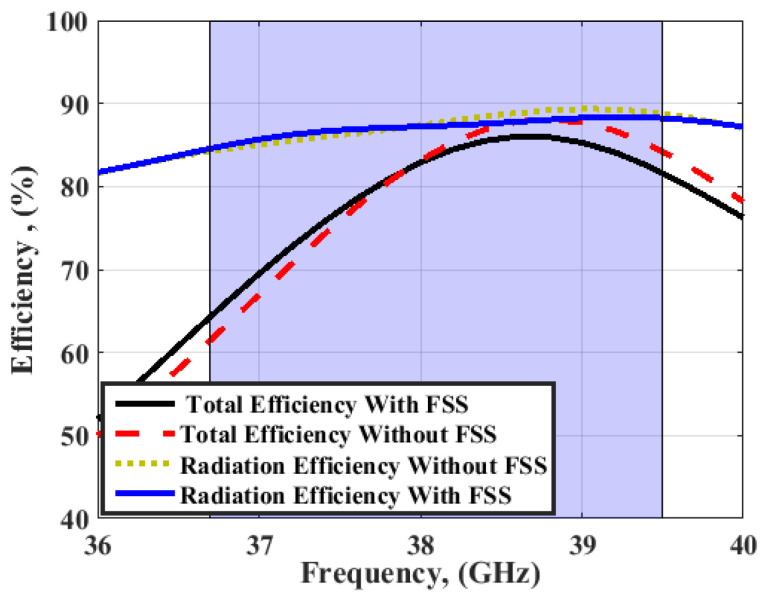
The simulated total and radiation efficiency of the MIMO antenna at port 1 with/without FSS.

The measured gain and radiation patterns are extracted by the technique discussed in [30,31]. It is seen that the antenna with FSS has simulated peak gain ranging from 8.2 dBi to 10 dBi and measured peak gain ranging from 8 up to 10 dBi within the working band, while it achieves simulated peak gain around 5.8 dBi and measuring peak gain ranging from 4.5 dBi to 5.5 dBi without using the FSS structure. It can be concluded that the FSS can increase the antenna gain by 4.5 dBi higher than the antenna gain without FSS. Figure 26 displays the simulated total efficiency and the radiation efficiency of the MIMO antenna at port 1 with/without FSS. The radiation and total efficiencies of the suggested antenna are around 87% and 82%, respectively.

The diversity parameters of the MIMO antenna with FSS such as ECC, DG, and CCL are measured to determine the performance of the antenna in the MIMO system. The ECC can be accounted for using S-parameters and radiation field patterns to quantify the multiple port efficiency [32].

(1) $$ECC=\rho_{e}=\left|\rho_{ij}\right|=\frac{{\left|S_{\mathrm{ii}}^{*}S_{\mathrm{ij}}+S_{\mathrm{ji}}^{*}S_{\mathrm{jj}}\right|}^{2}}{\left(1-\left({\left|S_{\mathrm{ii}}\right|}^{2}+{\left|S_{\mathrm{ji}}\right|}^{2}\right)\right)\overset{}{}\left(1-\left({\left|S_{\mathrm{jj}}\right|}^{2}+{\left|S_{\mathrm{ij}}\right|}^{2}\right)\right)}$$

(2) $$ECC=\rho_{e}=\frac{{\left|\int \int 4\Pi \left[F_{1}\left(\theta ,\varphi \right)•F_{2}\left(\theta ,\varphi \right)d\Omega \right]\right|}^{2}}{\int \int 4\Pi {\left|F_{1}\left(\theta ,\varphi \right)\right|}^{2}d\Omega \int \int 4\Pi {\left|F_{2}\left(\theta ,\varphi \right)\right|}^{2}d\Omega }$$

**Figure 27:**
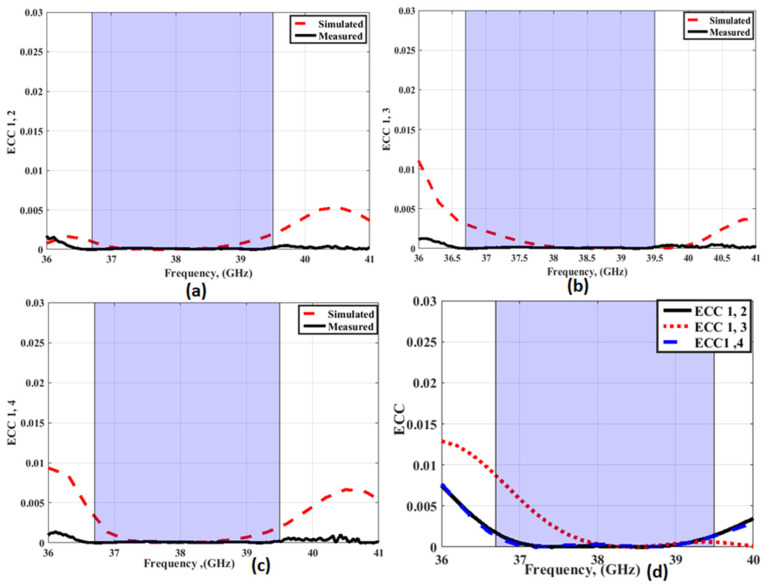
The ECC outcomes of the MIMO antenna with FSS at port 1 (**a**) ECC1, 2 (**b**) ECC 1, 3 (**c**) ECC 1, 4 (**d**) From radiation patterns.

An ECC < 0.5 is considered to be a good level for MIMO systems [33]. Figure 27a–c depict the ECC outcomes of the MIMO antenna with FSS at port 1 extracted from the S-parameters, and Figure 27d displays the ECC calculated from the radiation patterns as (2). The ECC values between ports 1 and 2; ports 1 and 3; and ports 1 and 4 are <0.005.

The DG can be calculated from ECC using Equation (3) [33].

(3) $$DG=10\times \sqrt{1-\left|ECC\right|}$$

**Figure 28:**
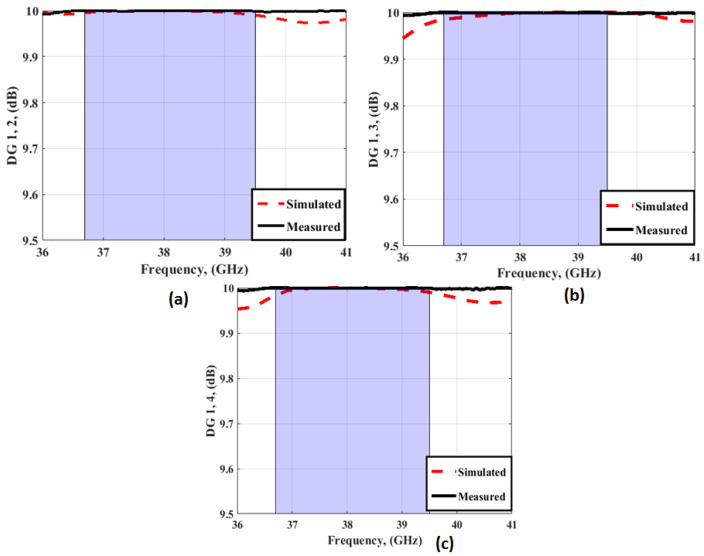
The DG outcomes of the MIMO antenna with FSS at port 1 (**a**) DG1, 2 (**b**) DG 1, 3 (**c**) DG 1, 4.

Figure 28 illustrates the DG outcomes of the MIMO antenna with FSS at port 1. The DG values between ports 1 and 2; between ports 1 and 3; and ports 1 and 4 are around 9.99, with a good trend between both outcomes.

The CCL indicates the upper constraint on data transmission rate and is studied to show its effect on the MIMO performance. The CCL should be <0.4 bits/s/Hz [32]. The CCL can be calculated using Equations (4) and (5) [34].

(4) $$C(Loss)=-\log_{2}\det(\psi^{R})$$

(5) $$\psi^{R}=\left[\begin{array}{cc} \rho 11 & \rho 12\\ \rho 21 & \rho 22 \end{array}\right],\rho_{ii}=1-\left({\left|S_{ii}\right|}^{2}+{\left|S_{ij}\right|}^{2}\right)\;and\;\rho_{ij}=-\left(S_{ii}^{*}S_{ij}+S_{ji}^{*}S_{ij}\right),\;for\;i,\;j\;=\;1\;or\;2$$

**Figure 29:**
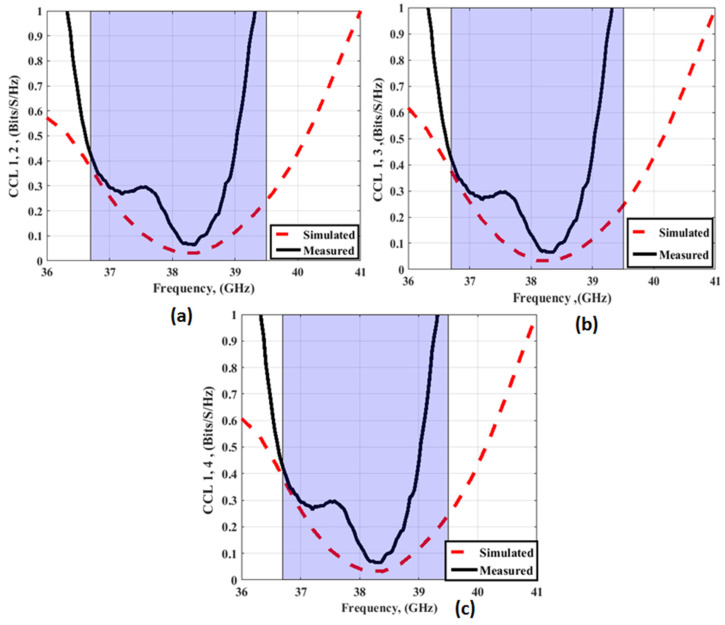
The CCL outcomes of the MIMO antenna with FSS at port 1 (**a**) CCL 1, 2 (**b**) CCL 1, 3 (**c**) CCL 1, 4.

Figure 29 shows the CCL outcomes of the MIMO antenna with FSS at port 1. The CCL values between ports 1 and 2; between ports 1 and 3; and ports 1 and 4 are <0.4 bit/s/Hz from 37 GHz to 39 GHz.

Table 1 tabulates the suggested antenna features in comparison to other reported designs. From Table 1, it is clear that the suggested MIMO achieved good results which recommended it to be utilized in the 5G networks.

## 6. Conclusions

For 5G communications, a four-element MIMO antenna with an FSS has been proposed. The proposed MIMO antenna was intended to be operated at frequency ranges from 37.2 GHz to 39.2 GHz, with isolation greater than 25 dB at the operating band. Gain enhancement has been achieved by employing an FSS compared to the MIMO configuration without an FSS structure. MIMO metrics such as ECC, DG, and CCL have been calculated from simulated and measured data to validate the diversity performance of the proposed antenna and to demonstrate its superior features. The simulated and measured data match well through the operated band, implying that the proposed structure can be recommended to be utilized in 5G communications.

## Figures and Tables

**Table 1:** The suggested antennas vs other designs.

Ref.	No of Elements	ε_r_/Thickness (mm)	Frequency [GHz]	B.W [GHz]	Gain (dB)/Efficiency (%)	Isolation (dB)	Gain Improvement Technique	Size (mm^2^)
[11]	4	2.2/0.381	28/38/38	2.4	7.6/(60–85)	≥17	-	156 × 77.8
[12]	4	2.2/0.787	27–30	3	6.1/84	≥28	-	30 × 30
[18]	4	2.92/1.027	26/28	6.3	7.1/90.7	≥17	Array	24.1 × 7
[19]	4	2.2/5.5	28	5	12.5/71.8	-	Array	47 × 41
[20]	8	2.2/0.254	32	11.5	12/93	-	Array	45 × 45
[21]	1	2.2/0.8	33	7	5.5/66.5	-	AMC	30 × 16
[22]	2	3.55/5	28	5.5	10/91	≥25	AMC	47 × 47
[24]	1	2.2/14	24.5	3	10.3/79.23%	-	FSS	40 × 40
[25]	2	(3) (2.2)/5	28	1	8.2/93%	≥30	FSS	35 × 25
[26]	4	(6.15) (2.2)/3.5	30	5	8/-	≥20	FSS	30 × 30
This work	4	3.55/5	38	3	8.2–10/82%	≥25	FSS	36.2 × 36.2

## Data Availability

All data generated or analyzed during this study are included in this article.
